# Meta-analysis of scar formation and depression and anxiety symptoms in patients after cardiac surgery

**DOI:** 10.3389/fpsyt.2025.1628394

**Published:** 2025-10-02

**Authors:** Yuting Wang, Wenyi Liu, Lixin Lin

**Affiliations:** ^1^ Department of Dermatology and Cosmetology Surgery, Yuhuangding Hospital, Yantai, China; ^2^ Department of Cardiovascular Surgery, Yantai Yuhuangding Hospital, Yantai, China; ^3^ Department of Burn and Plastic Surgery, Yantai Yuhuangding Hospital, Yantai, China

**Keywords:** Patient and Observer Scar Assessment, POSAS, meta-analysis, depression, anxiety, scars

## Abstract

**Background:**

The vertical median skin scar associated with median sternotomy, a standard approach for most cardiac surgeries, can cause psychological distress in patients, particularly depression and anxiety. The impact of scarring after cardiac surgery on depression and anxiety symptoms in patients is not well understood.

**Aim:**

The purpose of this meta-analysis was to investigate the effect of scarring on depressive and anxiety symptoms in patients after cardiac surgery.

**Methods:**

To investigate the relationship between scar formation and depression and anxiety symptoms in patients after cardiac surgery. We searched databases such as Web of Science, Cochrane Library, PubMed, and Embase for studies published before August 2024 on scar descriptions and psychological states after cardiac surgery. After data extraction and quality assessment, we used RevMan5.4 to analyze the depression and anxiety symptoms of patients after scar formation. Two authors independently performed the focused analyses and reached a final consensus on the included studies, which were subsequently quality checked and risk of bias assessed by a third author.

**Results:**

Four studies were included in the meta-analysis. All 4 studies used Patient and Observer Scar Assessment Scale (POSAS) to assess scar, and one study also combined Vancouver Scar Scale (VSS) for scar assessment. Meta-analysis results show that Full sternotomy has a smaller scar score than Limited sternotomy (OR = 0.94 [95% confidence interval (CI) 0.28-1.61]; P = 0.005), and there is no significant heterogeneity between the two groups (I^2^ = 0%). And the postoperative depression score in the Full sternotomy group was higher than that in the Limited sternotomy group (OR = 1.61 [95%CI 0.63-2.60]; P = 0.001), and there was no significant heterogeneity between the two groups (I^2^ = 0%). However, there was no statistical difference in postoperative anxiety scores between the two groups (OR = 0.70 [95%CI 1.40-2.80]; P = 0.51). There was slight heterogeneity between the two groups (I^2^ = 58%), so a random effects model was used.

**Conclusion:**

In conclusion, patients with more severe scarring after cardiac surgery may have more severe depressive symptoms, but adequately powered randomized controlled trials are needed to confirm these results.

## Introduction

Heart-related diseases are one of the leading causes of human death, and the incidence of heart disease reported by the World Health Organization in 2024 shows that the impact of such diseases is increasingly serious (30). According to research, cardiovascular disease (CVD) remains the leading cause of death worldwide, accounting for 32% of global deaths (3). The pathogenesis of heart disease is complex and involves multiple risk factors, including hypertension, diabetes, smoking, and poor eating habits (5, 29). Furthermore, heart disease-related mortality differs significantly between genders and geographic regions, suggesting the need for more effective prevention and intervention measures targeting specific populations and regions (31). The occurrence of heart disease is usually accompanied by reduced quality of life, disability, and premature death, which causes great harm to humans (10, 38).

The treatment of heart disease mainly relies on surgery, including coronary artery bypass surgery, total sternotomy and minimally invasive cardiac surgery. However, traditional heart surgery often leaves large scars after the operation. One study showed that patients who underwent median sternotomy developed significant scarring after surgery, which may lead to post-operative depression and anxiety (4). This association can be contextualized through several theoretical frameworks: Research indicates that sociocultural pressures and media-promoted body ideals significantly influence body image dissatisfaction. Through media-consumed body idealization and self-differentiation perception, individuals are prone to develop body image dissatisfaction (19, 25). Furthermore, social comparison and body monitoring are recognized as crucial mediating factors in the relationship between body ideal internalization and body image dissatisfaction (9, 26). These studies highlight the pivotal role of sociocultural elements in shaping body image dissatisfaction while underscoring the importance of social media as a source of appearance-related stress in modern society. For instance, studies show that scars from self-harm are strongly associated with negative body image perceptions, which may further exacerbate social withdrawal behaviors (7). Additionally, social stigma and self-criticism are recognized as crucial factors contributing to body image-related shame (8). Although patients have significant improvements in exercise capacity and quality of life after surgery, there may be differences in the specific performance of postoperative recovery among patients with different surgical methods (15). In addition, with the development of minimally invasive surgical technology, more and more studies have begun to focus on how to reduce scars and complications after surgery. This method not only reduces postoperative scars, but also maintains the stability of the chest and avoids the risks associated with total sternotomy (34). Therefore, choosing an appropriate surgical method can not only improve the surgical effect and reduce the formation of postoperative scars, but also reduce the patient’s psychological burden to a certain extent (21, 34).

To further clarify the effect of scar formation after cardiac surgery on anxiety and depression in patients, we conducted a systematic review and meta-analysis of studies on scar scores and anxiety and depression scores in patients after cardiac surgery to evaluate the effect of scar on anxiety and depression in patients after cardiac surgery.

## Materials and methods

### Search strategy

This meta-analysis evaluated all studies according to the Preferred Reporting Items for Systematic Reviews and Meta-Analyses (PRISMA) guidelines. All studies were reviewed independently by two authors. A systematic electronic literature search of Web of Science, Cochrane Library, PubMed, and Embase databases was conducted from inception to August 2024 using the following combined MeSH heading and keyword search terms. The search terms were combined from the following medical subject headings: “ cardiac surgical procedures, congenital heart disease, cardiac septal defect, atrial septal defect, mini-thoracotomy, thoracotomy, incision, cardiac surgery, coronary artery bypass grafting, CABG, cardiac surgery, valve replacement, or thoracic surgery, score, etc.” [AND] and [OR] were used as Boolean operators in the search strategy. The search was limited to clinical trials involving adult participants and published in English. The reference list of each full text used for the review and any existing reviews identified were manually searched to identify additional potentially eligible citations not found in the electronic database search.

### Inclusion criteria

To ensure selection of relevant studies, the following inclusion criteria were used. This included studies with scar assessment and measuring depression/anxiety scores as secondary outcomes in order to include more studies in areas that have been exhausted. Unpublished and ongoing studies were not identified and therefore excluded. The inclusion criteria for the current meta-analysis were as follows: participants: Patients who have undergone cardiac surgery; interventions: the full sternotomy and the limited sternotomy; comparators: scar formation and depression and anxiety symptoms.

### Data extraction and evaluation of quality

The literature was managed using Endnote X9 (Clarivate, London, UK), and studies that were irrelevant, duplicates, or non-experimental (especially those based on animals) were excluded. Following this, the titles and abstracts were carefully reviewed to eliminate studies that did not meet the inclusion criteria. The remaining literature was examined in detail, ensuring a thorough and systematic process. The full texts of the identified studies were reviewed for a secondary screening, and any disagreements were settled through consensus discussions among three researchers. Two researchers assessed the quality of the literature independently using the Cochrane bias tool.

### Statistical analysis

Meta-analysis was conducted using Review Manager version 5.4 (The Cochrane Collaboration). Effect sizes were calculated by converting measurement data into mean differences (MD), while count data were represented as odds ratios (OR). The heterogeneity among the included studies was assessed using Cochran’s Q statistic, and the Higgins I² test was employed to evaluate the variability of effect sizes. A statistically significant heterogeneity was indicated by an I² value exceeding 50%. To address any apparent heterogeneity, parallel subgroup and sensitivity analyses were performed. A fixed-effects model was applied for the meta-analyses when no significant heterogeneity was detected among the studies. A P-value of less than 0.05 was considered statistically significant.

### Risk of bias and certain of evidence

The quality of the articles and our research was evaluated using the Risk of Bias Tool 2 (RoB2) and GRADE approaches. The RoB2 tool focused on five critical areas: (i) the process of randomization, (ii) deviations from the intended interventions, (iii) missing outcome data, (iv) the measurement of outcomes, and (v) the selection of reported results. In cases of disagreement, the reviewers collaborated until they achieved a consensus.

## Results

### Study selection

**Figure 1:**
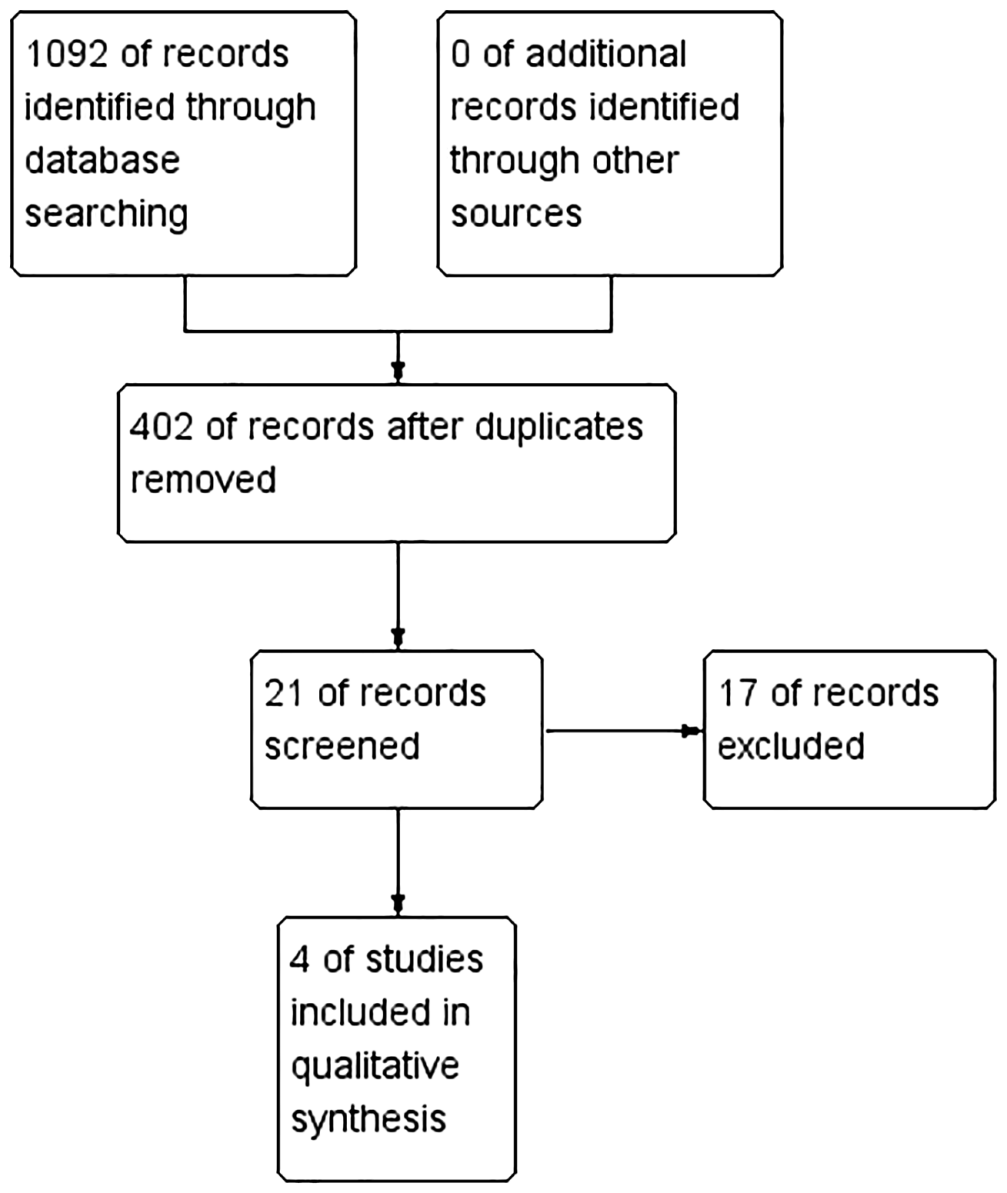
PRISMA flow diagram. PRISMA, preferred reporting items for systematic reviews and meta-analyses.

**Figure 1** shows the flowchart of the study selection process. After the initial review of titles and abstracts, 402 studies remained after removing duplicates. Upon further examination of the abstracts, 21 studies met the preliminary screening criteria, and ultimately, 4 studies (16, 18, 28, 37) were selected for analysis after reviewing the full texts again.

### Research characteristics

**Table 1** summarizes the characteristics of the studies included in this review.

**Table 1:** Summarizes the characteristics of the studies included in this review.

Publication		Kaya M.2015	İyigün T.2017	Piarulli A.2020	Sun KP.2020
No. of Studies		12	13	14	15
Type of study		NRCT	RCT	NRCT	NRCT
Region		Turkey	Turkey	Italy	China
Population		ASD	Underwent cardiac surgery	Heart valve disease	ASD
Incision scars		VSS,POSAS	POSAS	POSAS	ASD size
Total Cases		26	62	87	110
Full sternotomy	Surgical techniques	MS	Open group	CS	CS
	Scar score	14.6 ± 8.0	4.59 ± 1.38	20.4 ± 9.9	18.3 ± 4.7
	cases (n)	14	29	48	55
	Anxiety (Mean ± SD)	5.8 ± 4.8	5.79 ± 4.67	41.0 ± 11.8	8.27 ± 3.4
	Depression(Mean ± SD)	–	4.83 ± 3.68	9.8 ± 8.3	8.6 ± 3.8
Limited sternotomy	Surgical techniques	RLMT	Robotic group	MIVS	Percutaneous device closure group
	Scar score	13.5 ± 7.9	3.64 ± 1.52	17.1 ± 9.9	17.9 ± 5.4
	cases (n)	12	33	39	55
	Anxiety(Mean ± SD)	10.0 ± 7.7	5.58 ± 3.5	36.3 ± 10.2	7.05 ± 3.7
	Depression(Mean ± SD)	–	3.79 ± 3.18	6.1 ± 6.7	6.9 ± 3.4
Post-op measurement times		6-months after	6-months after	6-months after	1 year after
Anxiety and depression Screening Instrument		HAM-A	HAD	STAI-Y,BDI-II	HADS

SD, Standard deviation; RCT, Randomized Controlled Trial; NRCT, Nonrandomized controlled trial; ASD, Atrial septal defects; VSS, Vancouver Scar Scale; POSAS, Patient and Observer Scar Assessment; MS, Median sternotomy; CS, Conventional sternotomy; RLMT, Right lateral minithoracotomy; MIVS, Minimally invasive cardiac valve surgery; HAM-A, Hamilton Rating Scale for Anxiety; HAD, Hospital anxiety and depression scale; STAI-Y, State-Trait Anxiety Inventory Form Y; BDI-II, Beck Depression Inventory-II, HADS, Hospital anxiety and depression scale.

### Study methodology

**Figure 2:**
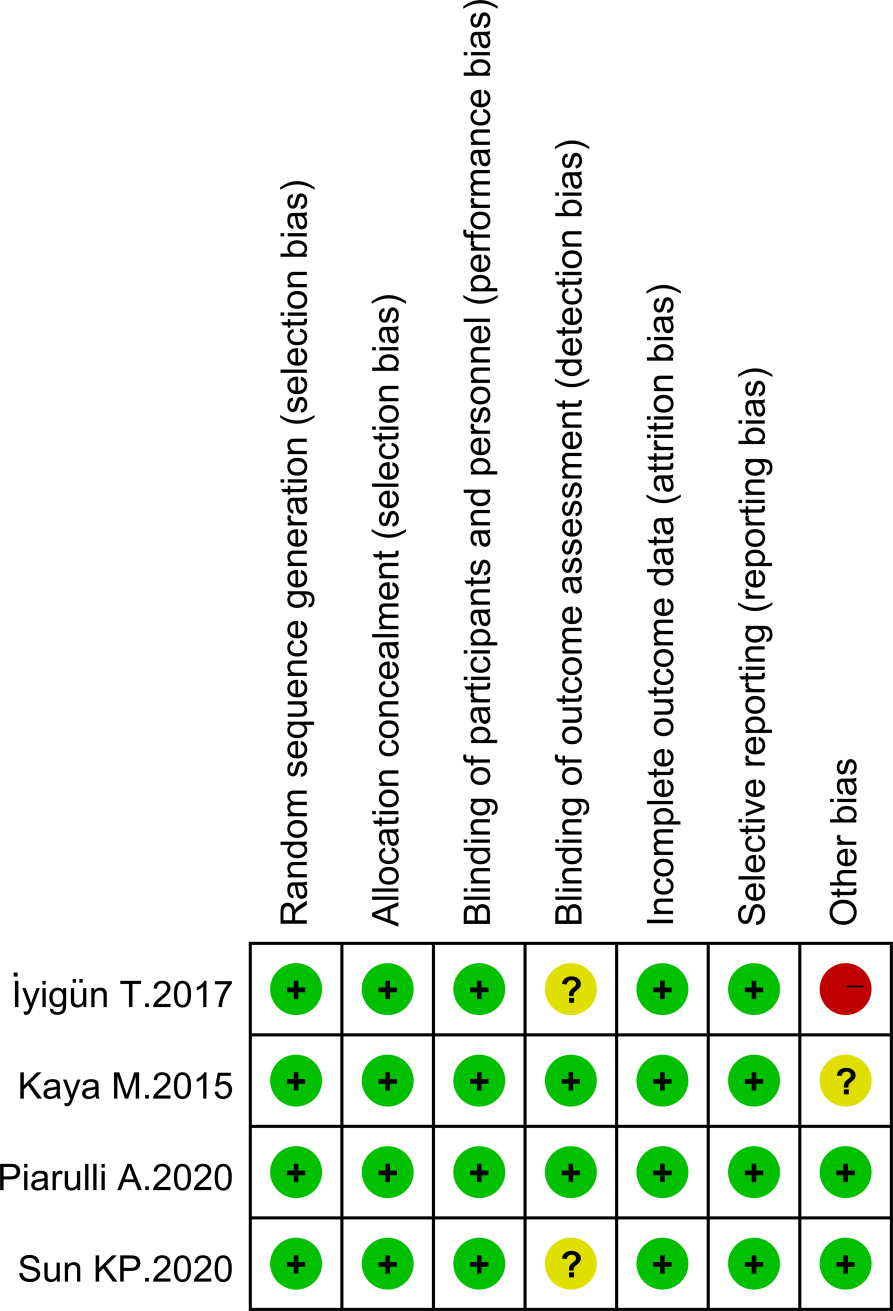
Risk of bias graph.

**Figure 3:**
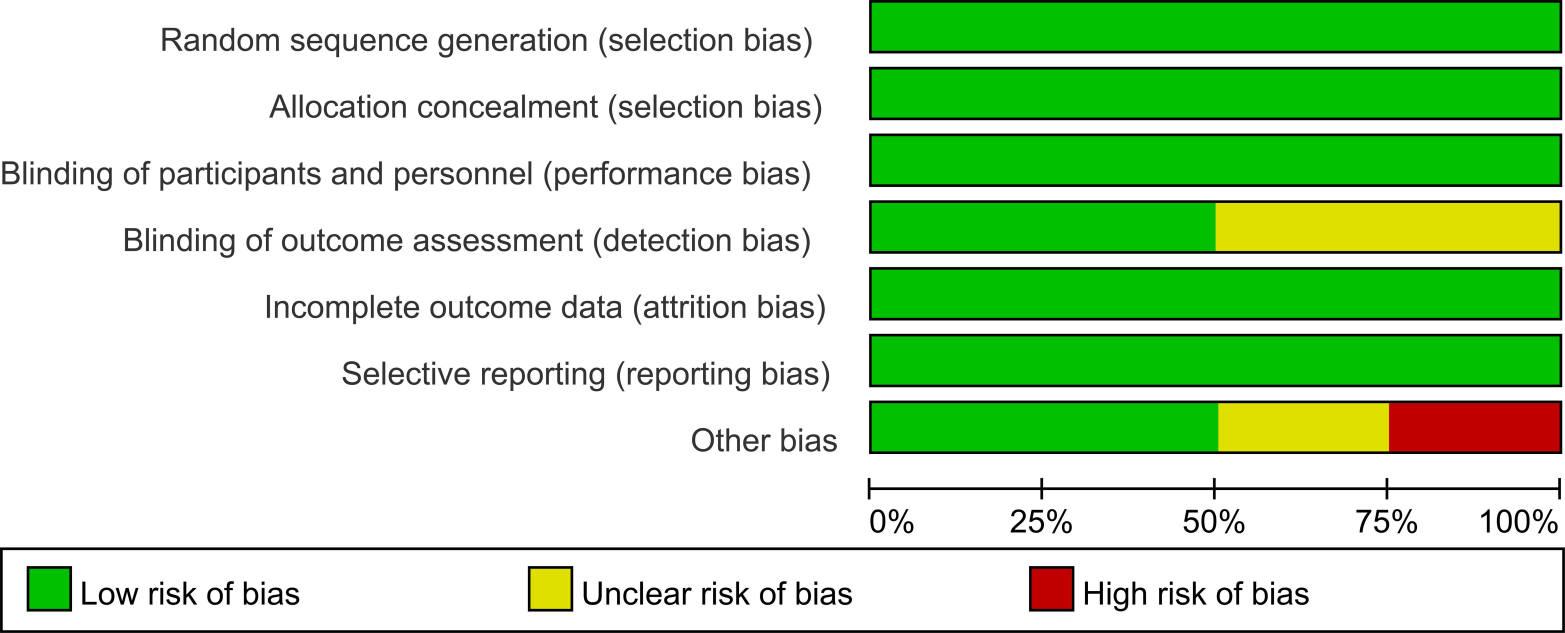
Risk of bias summary.

The analysis compared the baseline status of patients in four studies. **Figures 2**, **3** show tables outlining the “risk of bias in included studies.” A detailed description of the study features can be found in the section labeled “Features included in the study.”

### Comparison of scar size after cardiac surgery

**Figure 4:**
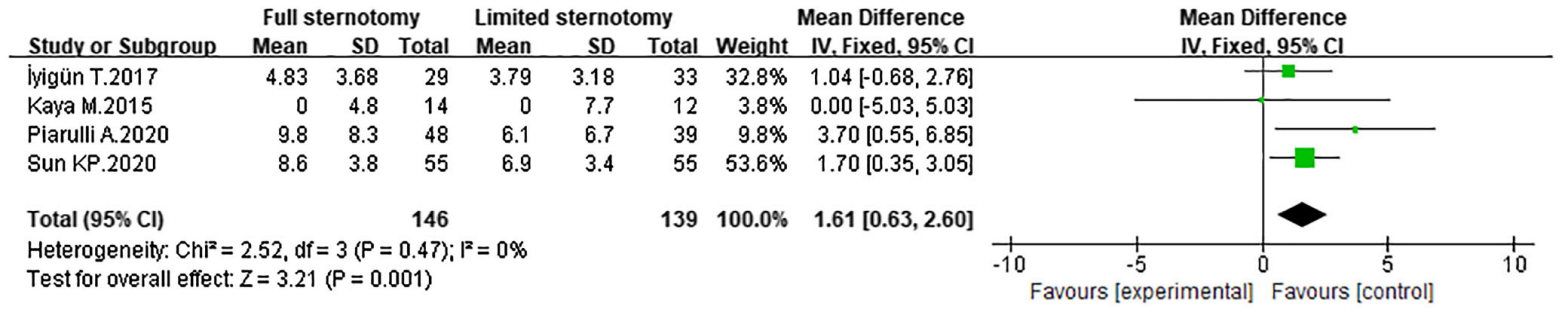
Forest map of scar comparison between the full sternotomy group and the limited sternotomy group after cardiac surgery.

All four studies compared the scores 6 months after cardiac surgery. There was no heterogeneity between the two groups of studies (I^2^ = 0%). The scar after cardiac surgery in the full sternotomy group was significantly smaller than that in the limited sternotomy group (OR 0.94 [95% CI 0.28-1.61]; P = 0.005) (see **Figure 4**).

### Comparison of depression scores between the two groups after cardiac surgery

**Figure 5:**
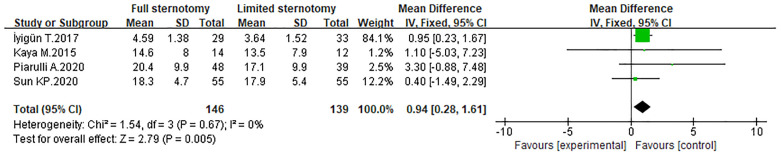
Forest map of depression scores between the full sternotomy group and the limited sternotomy group after cardiac surgery.

All four studies compared depression scores 6 months after cardiac surgery. There was no heterogeneity between the two groups (I^2^ = 0%). The depression score after cardiac surgery in the Full sternotomy group was significantly higher than that in the Limited sternotomy group (OR1.61[95%CI:0.63-2.60]; P = 0.001) (see **Figure 5**).

### Comparison of anxiety scores between two groups after cardiac surgery

**Figure 6:**
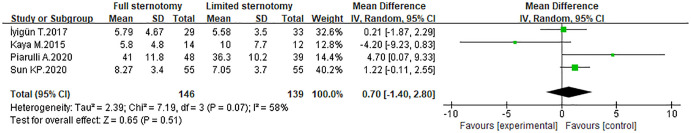
Forest map of anxiety scores between the full sternotomy group and the limited sternotomy group after cardiac surgery.

All four studies compared anxiety scores 6 months after cardiac surgery. There was slight heterogeneity between the two studies (I^2^ = 58%). Therefore, a random effects model was used. Compared with the Limited sternotomy group, the OR of post-cardiac surgery anxiety score in the Full sternotomy group was 0.70 [95% CI:1.40-2.80]; P = 0.51) (see **Figure 6**), which was not statistically significant difference.

### Publication bias analysis

**Figure 7:**
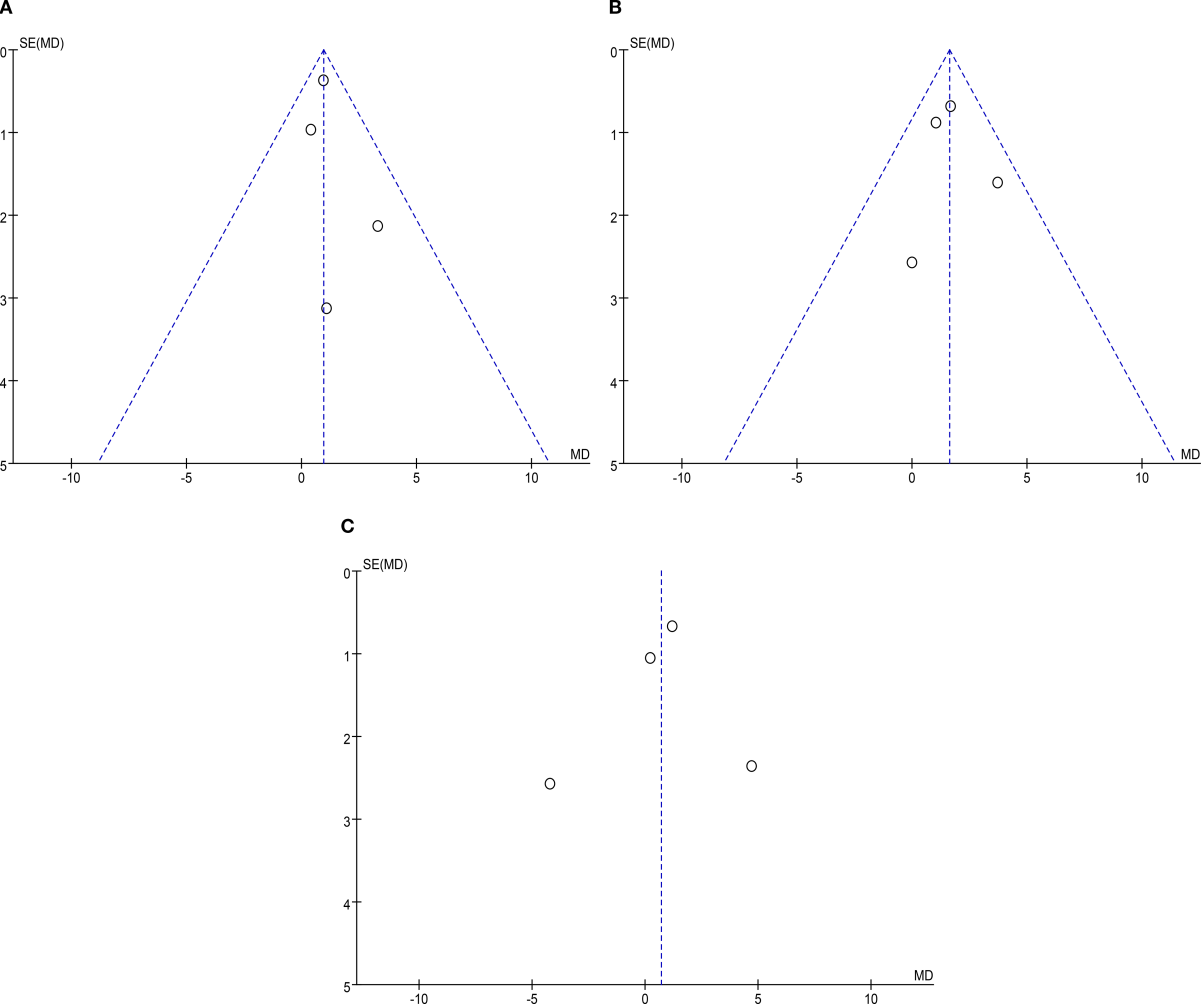
The funnel plots of each outcome. **(A)** Scar comparison; **(B)** Depression scores; **(C)** Anxiety scores.

Funnel plots were employed to evaluate publication bias for each outcome. As illustrated in **Figures 7A** through **7C**, the funnel plots exhibit good symmetry, with scatter points evenly distributed within the funnel. This suggests that publication bias is unlikely to significantly impact the results of the meta-analysis (see **Figure 7**).

## Discussion

Full sternotomy is a common method for treating heart disease, but patients often develop severe scars after surgery. Scar formation not only affects the patient’s physical recovery, but may also have a significant impact on his or her psychological state. Studies have found that chest scars, as the most obvious abnormal feature after surgery, not only affect the patient’s self-perception and self-esteem, but may also cause long-term mental health problems, such as anxiety and depression (1, 17, 22, 24). One study showed that visible chest scars may cause patients to feel low self-esteem, anxiety, or depression, especially in female patients and patients with obvious scars (such as hypertrophic scars). This psychological impact is more significant (20). Patients may avoid wearing certain types of clothing or participating in social activities due to the presence of scars, further exacerbating their psychological burden (33).

Compared with traditional full sternotomy, limited sternotomy has a smaller incision, shorter scar length after surgery, and may have less impact on patients’ anxiety and depression (12, 23, 27, 35). This meta-analysis included 285 patients who underwent cardiac surgery, 146 of whom underwent full sternotomy and 139 who underwent limited sternotomy. Full sternotomy had a smaller scar score than Limited sternotomy (OR = 0.94 [95% CI 0.28-1.61]; P = 0.005), with no significant heterogeneity between the two groups (I^2^ = 0%); and the postoperative depression score of Full sternotomy group is higher than that of Limited sternotomy group (OR = 1.61 [95%CI 0.63-2.60]; P = 0.001), there was no significant heterogeneity between the two groups (I^2^ = 0%). However, there was no statistical difference in postoperative anxiety scores between the two groups (OR = 0.70 [95%CI 1.40-2.80]; P = 0.51). There was slight heterogeneity between the two groups (I^2^ = 58%), so a random effects model was used. This is basically consistent with previous research results (6, 11, 36). The results of this study further illustrate that patients with more severe postoperative scars have higher depression scores, but there is no significant difference in anxiety. Possible reasons for analyzing the analysis are that depression is often associated with persistent negative self-evaluation and hopelessness, and may be more closely linked to the long-term, stable nature of scarring. In contrast, anxiety tends to be acute and is associated with immediate threats or uncertainties (e.g., surgical recovery complications, short-term social judgments) that may decrease as patients adjust to postoperative life, regardless of scar severity. In addition, the number of studies and patients included in this meta-analysis was relatively limited.

In order to reduce the impact of scarring after cardiac surgery on the psychological state of patients, the following measures will be taken in the clinic in the future: Psychological intervention: Provide psychological support and counseling to help patients better cope with the psychological pressure caused by postoperative scarring. Psychological intervention can be carried out in individual or group forms, including cognitive behavioral therapy, emotion management techniques, etc. (32). Physical therapy: Use physical methods to reduce scarring, such as pressure therapy, silicone patches, etc. These methods can improve the appearance of scars, thereby reducing patients’ anxiety and depression (14). Drug treatment: Use local drugs (such as corticosteroids) to reduce scar formation and improve its appearance, thereby reducing patients’ negative psychological reactions (13). Education and support: By educating patients about the process and treatment of scarring, their self-management and coping abilities can be enhanced. In addition, establishing patient support groups to allow patients to exchange experiences and feelings with each other can also help relieve psychological stress (2).

### Limitations

This meta-analysis has some limitations. Firstly, due to the limited number of studies on the effect of scar formation after cardiac surgery on postoperative anxiety and depression, this study only included 4 studies, and the sample size of each study was relatively small. Therefore, the relationship between scar formation after cardiac surgery and depression and anxiety needs to be further confirmed. Secondly, although the heterogeneity between the included studies was low, other potential factors may affect the summary results, such as gender, social support, and marital status. Notably, we did not account for Hypermobility Spectrum (defined by a Beighton score above 4), a known risk factor associated with both hypertrophic scars/keloids and significantly higher rates of anxiety (22-fold increased risk) and depression (over 3-fold increased risk). Given that approximately 15% of the general population meets this criterion, it is likely that some included patients had this comorbidity, which may have confounded the observed associations between scar formation and psychological symptoms. Future studies can consider multicenter prospective studies to obtain more diverse data and improve the external validity of the study. Thirdly, our inclusion strategy was relatively narrow, as we focused primarily on studies using the Patient and Observer Scar Assessment and Vancouver Scar Scale for scar evaluation, and specific instruments for anxiety and depression assessment. We excluded studies employing alternative scar metrics or other psychological scales. This may have limited the breadth of data and introduced potential selection bias, as different assessment tools may capture distinct aspects of scar characteristics and psychological states, affecting the generalizability of our findings. Future studies should adopt a broader inclusion strategy, incorporating diverse scar and psychological assessment instruments, and clearly justify any exclusions to ensure a more comprehensive synthesis of evidence. Fourthly, the follow-up time for scar formation in this study was 6 months after surgery, which is relatively short. Long-term follow-up studies can be carried out in the future to understand the long-term changes of scars on patients’ psychological state. At the same time, personalized psychological intervention programs can be designed in the future, which may be more effective in improving the mental health of patients after scar formation.

## Conclusion

The results of this meta-analysis show that compared with limited sternotomy, traditional full sternotomy has a larger incision, more obvious postoperative scars, and more severe postoperative depressive symptoms in patients, but it has no impact on postoperative anxiety. Not significant. Larger prospective studies of patients with postcardiac surgery scarring are warranted to better understand the relationship between postcardiac surgery scarring and symptoms of depression and anxiety.

## Data Availability

The raw data supporting the conclusions of this article will be made available by the authors, without undue reservation.
